# The World Federation of Neurosurgical Societies Young Neurosurgeons Survey (Part I): Demographics, Resources, and Education

**DOI:** 10.1016/j.wnsx.2020.100083

**Published:** 2020-10

**Authors:** Sujit Gnanakumar, Bilal Abou El Ela Bourquin, Faith C. Robertson, Davi J. Fontoura Solla, Claire Karekezi, Kerry Vaughan, Roxanna M. Garcia, Fahd Derkaoui Hassani, Alexander Alamri, Julius Höhne, Nesrine Mentri, Martin Stienen, Tsegazeab Laeke, Luis Rafael Moscote-Salazar, Ahmed Nasser Al-Ahmari, Hosam Al-Jehani, Federico Nicolosi, Nicolás Samprón, P. David Adelson, Franco Servadei, Ignatius N. Esene, Amro Al-Habib, Angelos G. Kolias, World Federation of Neurosurgical Societies Young Neurosurgeons Committee

**Affiliations:** 1 School of Clinical Medicine, University of Cambridge, Cambridge, United Kingdom; 2 National Institute for Health Research Global Health Research Group on Neurotrauma, University of Cambridge, Cambridge, United Kingdom; 3 Department. of Neurosurgery, Massachusetts General Hospital, Boston, Massachusetts, USA; 4 Department of Neurosurgery, University of São Paulo, Brazil; 5 Department of Neurosurgery, Rwanda Military Hospital, Kigali, Rwanda; 6 Department of Neurosurgery, University of Pennsylvania, Philadelphia, Pennsylvania, USA; 7 Department of Neurosurgery, Northwestern University, Chicago, Illinois, USA; 8 Department of Neurosurgery, Cheikh Zaid International Hospital, Abulcasis International University of Health Sciences, Rabat, Morocco; 9 Department of Neurosurgery, The Royal London Hospital, London, United Kingdom; 10 Department of Neurosurgery, University Medical Center Regensburg, Regensburg, Germany; 11 Department of Neurosurgery, Bejaia University Hospital, Bejaia, Algeria; 12 Department of Neurosurgery, University Hospital Zurich and Clinical Neuroscience Center, University of Zurich, Switzerland; 13 Department of Surgery, Neurosurgery Unit, Addis Ababa University, College of Health Sciences, Addis Ababa, Ethiopia; 14 Department of Neurosurgery, University of Cartagena, Cartagena de Indias, Colombia; 15 Division of Neurosurgery, Department of Neurosciences, King Faisal Specialist Hospital and Research Centre, Riyadh, Saudi Arabia; 16 Department of Neurosurgery, King Fahad Hospital of the University, Imam Abdulrahman bin Faisal University, Alkhobar, Saudi Arabia; 17 Neuroscience Center, King Fahad Specialist Hospital-Dammam, Dammam, Saudi Arabia; 18 Department of Neurosurgery, Humanitas University and Research Hospital, Rozzano, Milan, Italy; 19 Servicio de Neurocirugía, Hospital Universitario Donostia, San Sebastián, Spain; 20 Barrow Neurological Institute at Phoenix Children's Hospital, Phoenix, Arizona, USA; 21 Neurosurgery Division, Department of Surgery, University of Bamenda, Bamenda, Cameroon; 22 Division of Neurosurgery, Department of Surgery, King Saud University, Riyadh, Saudi Arabia; 23 Division of Neurosurgery, Department of Clinical Neurosciences, University of Cambridge and Addenbrooke's Hospital, Cambridge, United Kingdom; 24 World Federation of Neurosurgical Societies Central Office, Nyon, Vaud, Switzerland

**Keywords:** Demographics, Education, Global health, Global neurosurgery, Neurosurgery, Resources, Training

## Abstract

**Background:**

Providing a comprehensive and effective neurosurgical service requires adequate numbers of well-trained, resourced, and motivated neurosurgeons. The survey aims to better understand 1) the demographics of young neurosurgeons worldwide; 2) the challenges in training and resources that they face; 3) perceived barriers; and 4) needs for development.

**Methods:**

This was a cross-sectional study in which a widely disseminated online survey (April 2018–November 2019) was used to procure a nonprobabilistic sample from current neurosurgical trainees and those within 10 years of training. Data were grouped by World Bank income classifications and analyzed using χ^2^ tests because of its categorical nature.

**Results:**

There were 1294 respondents, with 953 completed responses included in the analysis. Of respondents, 45.2% were from high-income countries (HICs), 23.2% from upper-middle-income countries, 26.8% lower-middle-income countries, and 4.1% from low-income countries. Most respondents (79.8%) were male, a figure more pronounced in lower-income groups. Neuro-oncology was the most popular in HICs and spinal surgery in all other groups. Although access to computed tomography scanning was near universal (98.64%), magnetic resonance imaging access decreased to 66.67% in low-income countries, compared with 98.61% in HICs. Similar patterns were noted with access to operating microscopes, image guidance systems, and high-speed drills. Of respondents, 71.4% had dedicated time for neurosurgical education.

**Conclusions:**

These data confirm and quantify disparities in the equipment and training opportunities among young neurosurgeons practicing in different income groups. We hope that this study will act as a guide to further understand these differences and target resources to remedy them.

## Introduction

There is an urgent need to substantially increase the neurosurgical workforce as part of global surgical system strengthening to prevent death and disability for patients with neurologic disease. The global burden of neurosurgical disease is estimated to be 22.6 million patients per annum, of whom 13.8 million need therapeutic surgical intervention.^1^ Although there are an estimated 49,940 neurosurgeons worldwide, they are unequally distributed; neurosurgeon densities range from 0 to 58.95 (standardized to per 1 million population) between countries.^2^ In low-income countries (LICs) and middle-income countries, >5 million essential neurosurgical cases are not treated each year because of lack of access to services.^1^ These cases are mostly in sub-Saharan Africa and East Asia, where the ratio of neurosurgeon density to disease burden is critically low.^3^ It is estimated that 23,300 additional neurosurgeons are required to eliminate the operative deficit.^1^ In light of these geographic disparities, it is critical that neurosurgical staff are distributed according to population needs.

The global neurosurgical community has responded to the disparity by developing a consensus and putting neurosurgery on the global surgery and health policy agenda.^4^^,^^5^ The publication of the Lancet Commission “Global Surgery 2030” report inspired the neurosurgical community to create the field of global neurosurgery, defined by Park et al. (2016) as “an area for study, research, practice, and advocacy that places priority on improving health outcomes and achieving health equity for all people worldwide who are affected by neurosurgical conditions or have a need for neurosurgical care.”^6^^,^^7^ An international group of neurosurgeons convened to publish the Global Neurosurgery Consensus Document, which describes 7 areas required to expand access to neurosurgery worldwide, particularly in low-middle-income countries (LMICs): workforce, prehospital care, training and education, research, equipment, innovation, and advocacy (www.globalneurosurgery.org).

Training and education are critical in this effort to address neurosurgical inequities. Progress is being made in recruiting physicians, improving the number and quality of training programs, and retaining existing surgeons in their home nations. The Foundation for International Education in Neurological Surgery (www.fiens.org) and the World Federation of Neurosurgical Societies (WFNS; www.wfns.org) have spearheaded initiatives to train neurosurgeons in LMICs over the past decades. A plethora of other projects dedicated to building capacity are under way, such as Africa 100, the All India Institute of Medical Sciences Neurosurgery Education and Training School, and CURE International.^7^^,^^8^

Young neurosurgeons across the economic spectrum have different educational experiences and thus different needs because of variation in training programs, availability of academic opportunities, and access to equipment and expertise in local health systems. There is a paucity of studies that assess the needs of young neurosurgeons internationally. We surveyed the key needs of young neurosurgeons, their access to education and equipment, and the hurdles that they face in daily practice. This article highlights the demographics of young neurosurgeons and nuances in their access to training and equipment. Our goal is that the global neurosurgery community may use these insights to tailor context-specific interventions to the needs of our increasing neurosurgical workforce.

## Methods

### Survey Design and Dissemination

The WFNS Young Neurosurgeons Committee aims to represent and promote the interests of young neurosurgeons worldwide. The committee defines young neurosurgeons as residents, fellows, and consultants (within 10 years after the end of residency training). It aims to act as an advocate and conduit for developing the knowledge, surgical skills, research capability, and career opportunities for young neurosurgeons worldwide to align with the WFNS mission of benefiting patients and improving neurosurgical care.

The committee performed a cross-sectional study consisting of a self-administered survey, developed by the committee itself. Thirty open-ended multiple-choice questions (Appendix 1), assessed the following: survey respondents' demographics; the type of center in which they worked; access to imaging facilities and essential operating equipment; access to education and training; hurdles in daily practice; and the personal needs of trainees. We designed a concise survey to achieve high response rates and obtain the maximum amount of useful data possible. The survey was developed and piloted by members of the WFNS Young Neurosurgeons Committee and then approved by the leadership of the WFNS.

The Web-based survey link was distributed by the electronic mailing lists of continental, regional, national, and interest-based neurosurgical societies, e-mail to personal contacts, and social media platforms (Twitter, Facebook, and WhatsApp). The link directed the respondents to Qualtrics, where the survey could be completed online between April 25 and November 30, 2018.

### Inclusion and Exclusion Criteria

All survey responses by self-identified young neurosurgeons who completed all mandatory questions were included. All responses by nonyoung neurosurgeons or incomplete surveys were excluded.

### Statistical Analysis

Because of the wide dissemination of the questionnaire through social media platforms, calculation of a response rate was not possible. For descriptive purposes, categorical variables were presented with absolute and relative frequencies with estimated 95% confidence intervals. These were compared by means of the χ^2^ test for trend, considering the ordinal nature of the World Bank income groups stratification. Adjusted standardized residuals were analyzed when applicable.

All tests were 2-sided and final *P* values <0.05 were considered statistically significant. Multiple comparisons were implemented based on survey question structure. All analyses were conducted with SPSS version 24.0 for Windows (IBM Corp., Armonk, New York, USA).

## Results

The survey was completed by 1294 respondents and 953 completed surveys were included in the final analysis, representing a completion rate of 73.6%.

### Respondent Demographics and Scope of Clinical Practice

In terms of the World Bank country economic groups, 431 respondents (45.2%) were from high-income countries (HICs), 228 (23.9%) from upper-middle-income countries (UMICs), 255 (26.8%) from LMICs, and 39 (4.1%) from LICs. A complete list of respondents by World Bank classification is provided in Appendix 2.

The basic demographic data and scope of practice of survey respondents are shown in Table 1. There was no difference in age across economic groups. The largest cohort were those aged between 30 and 35 years, representing 40% of respondents. Significantly more respondents were male across all income groups, but this disparity was more pronounced among the lower-income group (*P* < 0.001). The HICs respondents tended to be less frequently based in areas with populations >1.5 million and more commonly in cities with between 50,000 and 1.5 million people (*P* < 0.001). Although only 33.9% of respondents in HICs were based in areas with populations >1.5 million, in LICs, this figure was 66.7%. Level of practice of respondents across the income groups was broadly similar for residents (41.3%) and fellows (10.5%). Consultants (attendings), those having completed neurosurgical training, with <5 years after the end of residency comprised more of the respondents in the lower-income groups. Most respondents (78.3%) regarded their job appointment as purely clinical and this was consistent across all income groups. Those from HICs and LICs were more likely to work only at university or teaching hospitals than were those from LMICs and UMICs, whose observed proportion at private and mixed private/public hospitals was higher (*P* < 0.001).

**Table 1:** Demographics and Scope of Practice

Variable	High-Income Economies (n = 431), n (%) (95% CI)	Upper-Middle-Income Economies (n = 228), n (%) (95% CI)	Lower-Middle-Income Economies (n = 255), n (%) (95% CI)	Low-Income Economies (n = 39), n (%) (95% CI)	Total (n = 953), n (%) (95% CI)	*P* Value
Age						0.931
<30 years	79 (18.3) (15–22.3)	42 (18.4) (13.9–24)	44 (17.3) (13.1–22.4)	9 (23.1) (12.7–38.3)	174 (18.3) (15.9–20.8)	
30–35 years	177 (41.1) (36.5–45.8)	96 (42.1) (35.9–48.6)	103 (40.4) (34.6–46.5)	16 (41) (27.1–56.6)	392 (41.1) (38.1–44.3)	
36–40 years	121 (28.1) (24–32.5)	64 (28.1) (22.6–34.2)	78 (30.6) (25.3–36.5)	9 (23.1) (12.7–38.3)	272 (28.5) (25.8–31.5)	
≥41 years	54 (12.5) (9.7–16)	26 (11.4) (7.9–16.2)	30 (11.8) (8.4–16.3)	5 (12.8) (5.6–26.7)	115 (12.1) (10.2–14.3)	
Female sex	124 (28.8) (24.7–33.2)	41 (18) (13.5–23.5)	26 (10.2) (7.1–14.5)	2 (5.1) (1.4–16.9)	193 (20.3) (17.8–22.9)	<0.001
Town/city population size						<0.001
>1.5 million	146 (33.9) (29.6–38.5)	136 (59.7) (53.2–65.8)	155 (60.8) (54.7–66.6)	26 (66.7) (51–79.4)	463 (48.6) (45.4–51.8)	
500,000–1.5 million	117 (27.2) (23.2–31.5)	42 (18.4) (13.9–24)	55 (21.6) (17–27)	7 (18) (9–32.7)	221 (23.2) (20.6–26)	
200,000–500,000	87 (20.2) (16.7–24.2)	21 (9.2) (6.1–13.7)	36 (14.1) (10.4–18.9)	4 (10.3) (4.1–23.6)	148 (15.5) (13.4–18)	
50,000–200,000	73 (16.9) (13.7–20.8)	21 (9.2) (6.1–13.7)	8 (3.1) (1.6–6)	2 (5.1) (1.4–16.9)	104 (10.9) (9.1–13.1)	
<50,000	8 (1.9) (1–3.6)	8 (3.5) (1.8–6.8)	1 (0.4) (0.1–2.2)	0 (0) (0–9)	17 (1.8) (1.1–2.8)	
Level of practice						0.016
Resident (<5 years after graduating from medical school)	98 (22.7) (19–26.9)	42 (18.4) (13.9–24)	40 (15.7) (11.7–20.7)	8 (20.5) (10.8–35.5)	188 (19.7) (17.3–22.4)	
Resident (≥5 years after graduating from medical school)	98 (22.7) (19–26.9)	51 (22.4) (17.4–28.2)	50 (19.6) (15.2–24.9)	7 (18) (9–32.7)	206 (21.6) (19.1–24.3)	
Fellow (additional training near the end or after the end of residency)	52 (12.1) (9.3–15.5)	14 (6.1) (3.7–10)	32 (12.6) (9–17.2)	2 (5.1) (1.4–16.9)	100 (10.5) (8.7–12.6)	
Consultant (<5 years after finishing residency)	106 (24.6) (20.8–28.9)	57 (25) (19.8–31)	89 (34.9) (29.3–40.9)	16 (41) (27.1–56.6)	268 (28.1) (25.4–31.1)	
Consultant (≥5 years after finishing residency)	72 (16.7) (13.5–20.5)	58 (25.4) (20.2–31.5)	40 (15.7) (11.7–20.7)	4 (10.3) (4.1–23.6)	174 (18.3) (15.9–20.8)	
Other	5 (1.2) (0.5–2.7)	6 (2.6) (1.2–5.6)	4 (1.6) (0.6–4)	2 (5.1) (1.4–16.9)	17 (1.8) (1.1–2.8)	
Job appointment type						0.471
Clinical	339 (78.7) (74.5–82.3)	179 (78.5) (72.7–83.4)	202 (79.2) (73.8–83.8)	26 (66.7) (51–79.4)	746 (78.3) (75.6–80.8)	
Clinical and academic	89 (20.7) (17.1–24.7)	47 (20.6) (15.9–26.3)	50 (19.6) (15.2–24.9)	13 (33.3) (20.6–49)	199 (20.9) (18.4–23.6)	
Research only	3 (0.7) (0.2–2)	2 (0.9) (0.2–3.2)	3 (1.2) (0.4–3.4)	0 (0) (0–9)	8 (0.8) (0.4–1.7)	
Main place of work						<0.001
University/teaching hospital	307 (71.2) (66.8–75.3)	124 (54.4) (47.9–60.7)	147 (57.7) (51.5–63.6)	27 (69.2) (53.6–81.4)	605 (63.5) (60.4–66.5)	
Other public hospital	77 (17.9) (14.5–21.8)	49 (21.5) (16.7–27.3)	26 (10.2) (7.1–14.5)	7 (18) (9–32.7)	159 (16.7) (14.5–19.2)	
Private hospital	21 (4.9) (3.2–7.3)	21 (9.2) (6.1–13.7)	41 (16.1) (12.1–21.1)	1 (2.6) (0.5–13.2)	84 (19.2) (7.2–10.8)	
Mixed public and private hospital	26 (6) (4.2–8.7)	34 (14.9) (10.9–20.1)	41 (16.1) (12.1–21.1)	4 (10.3) (4.1–23.6)	105 (11) (9.2–13.2)	

Summary of young neurosurgery respondents (n = 953) demographic characteristics and scope of clinical practice by World Bank income classification.

CI, confidence interval.

The most popular subspecialty interests were spinal surgery and neuro-oncology, followed by cerebrovascular surgery, neurotrauma, and skull base surgery (Table 2). Higher-income groups had a trend toward higher interest in neuro-oncology (*P* = 0.004), and less interest in neurotrauma (*P* = 0.018), cerebrovascular (*P* = 0.001), and skull base surgery (*P* = 0.013). Neuro-endoscopy interest as a subspecialty was higher for UMICs and LMICs than for HICs or LICs (*P* < 0.001). All income groups had on average >2 subspecialty interests, although the mean values for the UMICs (2.9 ± 1.8) and LMICs (2.8 ±1.8) were higher (analysis of variance post hoc tests, *P* < 0.001) than for the HICs (2.3 ± 1.4). The LICs had a mean number of subspecialty interests of 2.5 ± 1.7.

**Table 2:** Respondents Main Subspecialty Interests

Subspecialty	High-Income Economies (n = 431), n (%) (95% CI)	Upper-Middle-Income Economies (n = 228), n (%) (95% CI)	Lower-Middle-Income Economies (n = 255), n (%) (95% CI)	Low-Income Economies (n = 39), n (%) (95% CI)	Total (n = 953), n (%) (95% CI)	*P* Value
Cerebrovascular surgery	132 (30.6) (26.5–35.1)	99 (43.4) (37.2–49.9)	111 (43.5) (37.6–49.7)	16 (41) (27.1–56.6)	358 (37.6) (34.6–40.7)	0.001
Functional neurosurgery	69 (16) (12.9–19.8)	37 (16.2) (12–21.6)	51 (20.0) (15.6–25.3)	5 (12.8) (5.6–26.7)	162 (17) (14.8–19.5)	0.424
Neuroendoscopy	76 (17.6) (14.3–21.5)	69 (30.3) (24.7–36.5)	84 (32.9) (27.5–38.9)	8 (20.5) (10.8–35.5)	237 (24.9) (22.2–27.7)	<0.001
Neuro-oncology	196 (45.5) (40.8–50.2)	104 (36.5) (24.7–36.5)	93 (36.5) (30.8–42.5)	10 (25.6) (14.6–41.1)	403 (42.3) (39.2–45.5)	0.004
Neurotrauma	133 (30.9) (26.7–35.4)	102 (44.7) (38.4–51.2)	100 (39.2) (33.4–45.3)	15 (38.5) (24.9–54.1)	350 (36.7) (33.7–39.8)	0.018
Pediatric neurosurgery	80 (18.6) (15.2–22.5)	59 (25.9) (20.6–31.9)	61 (23.9) (19.1–29.5)	10 (25.6) (14.6–41.1)	210 (22) (19.5–24.8)	0.058
Skull base surgery	127 (29.5) (25.4–33.9)	78 (34.2) (28.4–40.6)	96 (37.7) (31.9–43.7)	16 (41) (27.1–56.6)	317 (33.3) (30.3–36.3)	0.013
Spinal surgery	167 (38.8) (34.3–43.4)	110 (48.3) (41.9–54.7)	119 (46.7) (40.6–52.8)	16 (41) (27.1–56.6)	412 (43.2) (40.1–46.4)	0.065
Other	19 (4.4) (2.8–6.8)	7 (3.1) (1.5–6.2)	9 (3.5) (1.9–6.6)	1 (2.6) (0.5–13.2)	36 (3.8) (2.7–5.2)	0.417

Young neurosurgery responses on main subspecialty interests by World Bank income classification (n = 953).

CI, confidence interval.

### Neurosurgical Services–General Characteristics and Availability of Key Features

The survey results regarding general neurosurgical services characteristics and availability of resources are summarized in Table 3. The respondents from HICs tended to work in hospitals with more beds, especially in the >1000-bed category (*P* < 0.001). Also, a higher-income group was associated with higher proportions of dedicated neurosurgical wards (*P* < 0.001). Twenty-five to 50 neurosurgical bed units were the most common type (37.8%), with <10% of centers having >100 beds. No centers in LICs had >100 beds.

**Table 3:** Responses to Questions Regarding Access to Space, Equipment, and Services

Variables	High-Income Economies (n = 431), n (%) (95% CI)	Upper-Middle-Income Economies (n = 228), n (%) (95% CI)	Lower-Middle-Income Economies (n = 255), n (%) (95% CI)	Low-Income Economies (n = 39), n (%) (95% CI)	Total (n = 953), n (%) (95% CI)	*P* Value
Number of hospital beds						<0.001
≤500	104 (24.1) (20.3–28.4)	111 (48.7) (42.3–55.1)	116 (45.5) (39.5–51.6)	18/39 (46.2) (31.6–61.4)	349 (36.6) (33.6–39.7)	
500–1000	173 (40.1) (35.6–44.8)	78 (34.2) (28.4–40.6)	75 (29.4) (24.2–35.3)	18 (46.2) (31.6–61.4)	344 (36.1) (33.1–39.2)	
>1000	154 (35.7) (31.4–40.4)	39 (17.1) (12.8–22.5)	64 (25.1) (20.2–30.8)	3 (7.7) (2.7–20.3)	260 (27.3) (24.6–30.2)	
Dedicated neurosurgical wards	371 (86.1) (86.1–89)	163 (71.5) (65.3–77)	184 (72.2) (66.4–77.3)	25 (64.1) (48.4–77.3)	743 (78) (75.2–80.5)	<0.001
Number of neurosurgical beds						0.966
<25	97 (22.5) (18.8–26.7)	77 (33.8) (28–40.1)	70 (27.5) (22.3–33.2)	17 (43.6) (29.3–59)	261 (27.4) (24.7–30.3)	
25–50	182 (42.2) (37.7–46.9)	83 (36.4) (30.4–42.8)	82 (32.2) (26.7–38.1)	13 (33.3) (20.6–49)	360 (37.8) (34.8–40.9)	
50–75	89 (20.7) (17.1–24.7)	30 (13.2) (9.4–18.2)	44 (17.3) (13.1–22.4)	7 (18) (9–32.7)	170 (17.8) (15.5–20.4)	
75–100	35 (8.1) (5.9–11.1)	18 (7.9) (5.1–12.1)	24 (9.4) (6.4–13.6)	2 (5.1) (1.4–16.9)	79 (8.3) (6.7–10.2)	
>100	28 (6.5) (4.5–9.2)	20 (8.8) (5.8–13.2)	35 (13.7) (10–18.5)	0 (0) (0–9)	83 (8.7) (7.1–10.7)	
Equipment and services access						
Computed tomography	426 (98.8) (97.3–99.5)	225 (98.7) (96.2–99.6)	250 (98) (95.5–99.2)	39 (100) (91–100)	940 (98.6) (97.7–99.2)	0.690
Magnetic resonance imaging	425 (98.6) (97–99.4)	198 (86.8) (81.8–90.6)	229 (89.8) (85.5–92.9)	26 (66.7) (51–79.4)	878 (92.1) (90.3–93.7)	<0.001
Catheter angiography	389 (90.3) (87.1–92.7)	149 (65.4) (59–71.2)	141 (55.3) (49.2–61.3)	4 (10.3) (4.1–23.6)	683 (71.7) (68.7–74.4)	<0.001
Operating microscope	427 (99.1) (97.6–99.6)	212 (93) (88.9–95.6)	212 (83.1) (78.1–87.2)	24 (61.5) (45.9–75.1)	875 (91.8) (89.9–93.4)	<0.001
Image guidance system (navigation)	388 (90) (86.8–92.5)	96 (42.1) (35.9–48.6)	86 (33.7) (28.2–39.7)	5 (12.8) (5.6–26.7)	575 (60.3) (57.2–63.4)	<0.001
High-speed drill	423 (98.1) (96.4–99.1)	198 (86.8) (81.8–90.6)	186 (72.9) (67.2–78)	17 (43.6) (29.3–59)	824 (86.5) (84.1–88.5)	<0.001
Intensive care unit	429 (99.5) (98.3–99.9)	225 (98.7) (96.2–99.6)	245 (96.1) (92.9–97.9)	36 (92.3) (79.7–97.4)	935 (98.1) (97–98.8)	<0.001
Mechanical ventilators in the intensive care unit	409 (95.3) (92.9–97)	222 (98.7) (96.2–99.6)	235 (95.9) (92.7–97.8)	35 (97.2) (85.8–99.5)	901 (96.4) (95–97.4)	0.083
Rehabilitation specialists	397 (92.1) (89.2–94.3)	176 (77.2) (71.3–82.2)	178 (69.8) (63.9–75.1)	19 (48.7) (33.9–63.8)	770 (80.8) (78.2–83.2)	<0.001

Summary of young neurosurgery respondents (n = 953) as it relates to access to space, equipment, and services by World Bank income classification.

CI, confidence interval.

Access to equipment and services highlights some significant differences between high-income and low-income settings. Access to computed tomography (CT) scanners and mechanical ventilators in the intensive care unit (ICU) were near universal (98.6% and 96.4%, respectively) and without significant differences across income groups. All other surveyed resources were less accessible the lower the country income (all *P* < 0.001). Whereas 98.6% had magnetic resonance imaging (MRI) access in HICs, this percentage decreased to 66.7% in LICs. There was access to catheter angiography for 90.3% of HICs respondents, but for only 10.3% of LICs respondents. Similarly, access to operating microscopes, image guidance systems, and high-speed drills was >90% in HICs but decreased to as low as 12.8% in the case of image guidance systems in LICs. Although there was widespread access to ICU beds across all income groups, with approximately 100% having access in HICs, almost 10% lacked access in LICs. A total of 92.1% of respondents had access to specialists in rehabilitation in HICs, but this statistic was as low as 48.7% in LICs.

### Education and Training

Questions ascertaining dedicated time for neurosurgical education are shown in Table 4. Most respondents (71.4%) had education opportunities, with a higher frequency reported by individuals in LMICs and LICs (78.43% and 76.92%, respectively; *P* = 0.006). In contrast, 68.2% of HICs respondents and 68.4% of UMICs respondents had dedicated teaching. Around half of the respondents in HICs and LMICs had a journal club held in their department, compared with approximately 30% of those in UMICs and LICs (*P* = 0.015). Across all groups, there was limited access (17.8%) to regular hands-on cadaveric training courses. This figure was lowest for the UMICs group (8.3%; *P* = 0.008). Of the respondents, 77.3% were members of national neurosurgical societies, which was most concentrated in HICs, where 82.8% were members; in contrast, only 66.7% were members in LICs (*P* = 0.005). Most respondents (60.0%) reported never having attended a WFNS conference or WFNS-supported meeting. This figure was significantly greater in HICs, where 68.7% had never attended a WFNS conference or supported meeting, compared with greater attendance frequency among the lower-income group (*P* < 0.001).

**Table 4:** Responses Pertaining to Training and Education

Questions	High-Income Economies (n = 431), n (%) (95% CI)	Upper-Middle-Income Economies (n = 228), n (%) (95% CI)	Lower-Middle-Income Economies (n = 255), n (%) (95% CI)	Low-Income Economies (n = 39), n (%) (95% CI)	Total (n = 953), n (%) (95% CI)	*P* Value
Do you have time dedicated for neurosurgical education	294 (68.2) (63.7–72.4)	156 (68.4) (62.1–74.1)	200 (78.4) (73–83)	30 (76.9) (61.7–87.4)	680 (71.4) (68.4–74.1)	0.006
Do you have a departmental journal club?	224 (52) (47.3–56.7)	70 (30.7) (25.1–37)	123 (48.2) (42.2–54.4)	11 (28.2) (16.6–43.8)	428 (44.9) (41.8–48.1)	0.015
Do you have access to regular hands-on cadaveric training courses in your department?	103 (23.9) (20.1–28.2)	19 (8.3) (5.4–12.6)	40 (15.7) (11.7–20.7)	8 (20.5) (10.8–35.5)	170 (17.8) (15.5–20.4)	0.008
Are you a member of a national neurosurgical society	357 (82.8) (79–86.1)	159 (69.7) (63.5–75.3)	195 (76.5) (70.9–81.3)	26 (66.7) (51–79.4)	737 (77.3) (74.6–79.9)	0.005
Attended a WFNS conference or a WFNS-supported meeting before						
Never	296 (68.7) (64.2–72.9)	117 (51.3) (44.9–57.7)	136 (53.3) (47.2–59.4)	18 (46.2) (31.6–61.4)	567 (59.5) (56.4–62.6)	<0.001
Once	77 (17.9) (14.5–21.8)	76 (33.3) (27.5–39.7)	69 (27.1) (22–32.8)	12 (30.8) (18.6–46.4)	234 (24.6) (21.9–27.4)	
Twice	25 (5.8) (4–8.4)	16 (7) (4.4–11.1)	23 (9) (6.1–13.2)	2 (5.1) (1.4–16.9)	66 (6.9) (5.5–8.7)	
More than 2 times	33 (7.7) (5.5–10.6)	19 (8.3) (5.4–12.6)	27 (10.6) (7.4–15)	7 (18) (9–32.7)	86 (9) (7.4–11)	

Summary of young neurosurgeons survey (n = 953) as it relates to training and education by World Bank income classification.

CI, confidence interval; WFNS, World Federation of Neurosurgical Societies.

## Discussion

This international survey is the most up to date and to our knowledge the most comprehensive study of the global practice and perspectives of young neurosurgeons. Because nearly 1000 complete responses were obtained from a distribution of HICs, UMICs, LMICs, and LICs, these data provide a cross-sectional look at the state of the field and elucidate opportunities for investment and improvement in efforts to meet the 2030 goals for mitigating the global burden of neurosurgical disease.

### Demographics

The clustering of respondents and, by inference, concentration of neurosurgical centers shed light on the rural–urban divide; it highlights that neurosurgeons in LICs were more likely to be based in larger urban areas, with few found in smaller towns and rural regions. Combined with factors such as poor transport infrastructure and access to the urban centers in which these neurosurgeons are based, people in rural areas have more limited access to neurosurgical care than do their urban counterparts. Factors such as time to intervention and access to trained personnel are strong determinants of mortality and poor outcome. Hence, training and access to equipment, as well as retention of specialists in rural areas, are critical to outcomes. Alternative approaches including telemedicine, mobile neurosurgical units, and training of other specialists in emergency neurosurgery should be carefully considered as neurosurgical capacity is strengthened. Other styles of care including partnerships with neurosurgeons based in large urban centers should also be explored.^9^

Nearly 80% of respondents identified as male, with the result being only slightly more pronounced in lower-income settings. This finding is consistent with the literature, which suggests that <6% of practicing neurosurgeons in North America are female.^10^ However, the representation of women in training programs is more numerous than in those neurosurgeons well into their careers. Efforts are under way to address this stark disparity, in particular the Women in Neurosurgery organization. Women in Neurosurgery was founded in 1989 and has strived to identify barriers such as lack of female mentors, unconscious biases, harassment, and salary inequalities and solutions to mitigate them.^11^ Barriers to practice for female neurosurgeons seem to have decreased within Europe.^12^ Less literature exists on the barriers faced by female neurosurgeons in other parts of the world and in LICs and how they may be overcome.

### Resources/Capacity

The survey shows marked disparities in resource distribution across country income groups. Encouragingly, nearly all survey respondents reported having access to a CT scanner (98.6%). CT scans are an essential tool in neurosurgery, particularly for diagnosis and prognosis in neurotrauma or acute stroke that may require emergency intervention.^13^ Delays in acquiring the scan may lead to worse patient outcomes.^14^^,^^15^ Although novel, portable, affordable technologies are being trialed for low-resource settings (e.g., handheld near-infrared spectroscopy devices for traumatic intracranial hematomas), CT scans remain the gold standard for rapid and accurate head imaging in neurotrauma. Multiple studies have also shown that the advent of CT imaging reduces mortality in the setting of central nervous system infections, which are more frequent in lower-income settings.^16^, ^17^, ^18^, ^19^ Although the reported rate of CT access was high, there is likely bias in the survey respondents being in urban settings such as university teaching hospitals, and there is likely still a need for scaling up access to CTs in less populated areas. In addition, the survey did not qualify whether access to scanning was consistent or not. The recently published *Comprehensive Policy Recommendations for Head and Spine Injury Care in LMICs* discussed the importance of having CT access as part of a neurotrauma center within 4 hours from 80% of the population. Access to MRI was also correlated to income group, with only 67% of LICs respondents reporting access. Neurosurgeons rely on MRI for higher-resolution definition of intracranial diseases such as tumors, stroke, infection, vascular anomalies, and soft tissue injuries within the spine. Although it may not be economically feasible to implement MRI technology in all hospitals in which neurosurgical care is delivered, having appropriate referral networks for MRI is needed.

Approximately 90% of HICs respondents reported access to angiography, compared with 10% in LICs. The global burden of stroke is a strong impetus to invest in interventional stroke treatment with angiography. The 2013 Global Burden of Disease study showed that stroke was the second most common cause of deaths (11.8% of all deaths) worldwide, after ischemic heart disease (14.8% of all deaths), and the third most common cause of disability.^20^ The study illuminated a concerning significant increase in stroke-related disability-adjusted life years and deaths in developing countries, but not developed countries, likely secondary to increasing metabolic and other noncommunicable diseases in these countries.^21^ Aneurysmal subarachnoid hemorrhage (aSAH) also faces a geographic mismatch between disease burden and treatment availability. In a 2018 meta-analysis of aSAH that included 58 studies from 31 different countries, Hughes et al. found a wide variation of aSAH across WHO regions from 0.71 to 12.38 per 100,000 persons, with almost two thirds of the burden in LMICs.^22^ Although the key to reducing the global burden of stroke is more effective prevention (reduction of hypertension, hyperlipidemia, diabetes, and smoking), the continual increase in stroke incidence argues for adding angiography to the armamentarium of more hospitals and neurosurgeons worldwide, backed by effective systems to allow patients to access these services in a timely manner.

Other operative tools and equipment were also lacking in lower-resource settings. Although there was >90% access to operating microscopes, image guidance systems, and high-speed drills in HICs, access to these tools decreased as low as 12.82% in LICs. These tools are critical to the safe and effective practice of neurosurgery, especially microneurosurgery, and if unavailable inherently limit a neurosurgical practice to more basic treatments, care, and training.

The results regarding access to ICU indicated that >90% of respondents had access to ICU beds across all income groups. Nevertheless, access does not necessarily mean that access is adequate and consistent for all patients who may need it.^23^ This finding may also reflect on what is defined as an ICU bed, because this is likely to vary regionally. In addition, ICU beds are often shared with other specialties, making the access limited or inadequate in some cases. Surgeries are frequently postponed because the limited critical care beds are usually occupied. In general, it is accepted that critical care capacity is limited in many LMICs and this issue was also highlighted as a barrier in the second part of the survey (see Part II).

### Education and Training

Surgical education has traditionally been dispensed in an apprenticeship-based model, the Halstedian “see one, do one, teach one” approach, by which younger surgeons are taught the ropes under the watchful guidance of their more experienced teachers, typically in the live operating theater.^24^ However, as surgery has become more subspecialized, coupled with restrictions on working hours in Europe and North America, case numbers performed during neurosurgical training seem to be declining.^25^^,^^26^ Focus has shifted toward other modalities of teaching including didactic and simulation teaching.^27^, ^28^, ^29^ Dedicated time for neurosurgical education is important for trainees to develop their skills outside the operating theater. Recent data suggest that the perceived quality of training directly influences the theoretic and practical skills set obtained by a resident at time of board certification.^30^ Although this question probed at the availability of dedicated learning time, it did not expand on how much protected time was available and what constituted “dedicated neurosurgical education,” which requires further elucidation.

Cadaveric training offers an opportunity to complement learning in the theater and can improve the trainees' anatomic knowledge and provide an opportunity to practice surgical techniques.^31^ Our results suggest low use (17.8%) of cadaveric training across the board. The results obtained in the current sample mirror the generally low to moderate satisfaction rate (about 22%) with the availability of opportunities for cadaveric training, documented by 532 trainees from Europe.^32^ This situation may be because of the costs associated with setting up and running a cadaveric training laboratory as well as questions as to whether cadaveric training is the most effective methods of training.^33^^,^^34^ Ethical and religious concerns in some states may mean that legal frameworks do not exist for the provision of cadaveric simulation. There exist other teaching aids including three-dimensional simulation tools that may also be equally or more effective compared with cadaveric training, albeit with their associated costs, which may be prohibitive in lower-income areas. This survey does not feature the use of other training modalities, which will be an important area of future work.^35^ Among European trainees in 2015, only about 12% expressed satisfaction with the options for simulator teaching to enhance their training.^32^ However, with technological advances and increasing implementation of simulators in the training programs of the (inter)national neurosurgical societies, their availability (at least in HICs and UMICs) has likely increased to some extent over the last few years. Another facet not explored in this survey is collaboration between neurosurgeons in HICs and LICs. Future work should look to characterize existing partnerships and the scope for developing new ones.

### Limitations

Because the survey was disseminated using electronic mailing lists of various neurosurgical societies, e-mail to personal contacts, and social media platforms, there was no way to ascertain response rate. In addition, those without access to reliable Internet, electronic devices, and e-mail are less likely to be captured in the study. The survey was administered in English, which limits respondents to those who are English speakers. Future region-specific or country-specific studies may want to translate surveys into local languages. There is also a strong likelihood of clustering of results with multiple respondents from the same institution. However, this situation is also indicative of the nature of neurosurgical practice, with multiple surgeons often clustered at a few large centers. The role played by academic and research contacts in dissemination of the survey may have affected the background of the respondent, particularly regarding the question enquiring whether the individual was paid solely for clinical work and/or research. More than 20% of respondents reported that they were paid to undertake some form of research, but this figure may be higher than that in the neurosurgical community in general because of the nature of the distribution of the survey. Further studies are required to corroborate and validate this result. Furthermore, most respondents were reporting from an urban setting, so ongoing practices and resources in rural or remote parts of these countries remain to be elucidated. This requirement is critical given the clear access inequity for rural populations and need for additional neurosurgical care.

These subjective needs and requests from countries should be interpreted with caution in their generalization to global settings. Although this survey provides a reference for resource strategies, partnership development, and system improvement, there will still be country-specific and hospital-specific needs that will have to be addressed on a more individualized basis. The social, political, and educational challenges that limit access to neurosurgical care should be assessed at a country-specific and region-specific level to understand unique factors.

The scope of our survey is limited to studying demographics and access to imaging, equipment, education, and training, as well as hurdles and personal needs. Other systems such as ancillary staff, anesthesia, and supply chains, which are part and parcel of neurosurgical care, have not yet been studied. Although there have been previous ground-level surveys of these resources by many national agencies, there are none specific to global neurosurgery. Questions about surgical equipment and resources assume that these are accessible, functional, and affordable and that surgeons are proficient in their use, which may not be true. We did not define or quantify access, which limits our interpretation of barriers facing young neurosurgeons to their own perspective, and not a quantifiable study of logistical barriers.

## Conclusions

With nearly 1000 participants, this survey is the most comprehensive understanding of the demographic characteristics of young neurosurgeons and the challenges that they face in their daily practice and development. We confirmed differences depending on the economic locality within which they practice. In LICs, young neurosurgeons have limited access to equipment and training modalities that are usually more widely available, albeit not extensively exploited, in high-income settings. We hope that these results will drive more detailed studies into the demographic, equipment, and training disparities that exist. Furthermore, we hope that the national health planners and the global neurosurgical community pay heed to these disparities and strive to ameliorate them through encouraging female participation and access to training, education, and equipment.

## Declaration of Competing Interest

A.G.K. is supported by the 10.13039/501100000272National Institute for Health Research (NIHR) Global Health Research Group on Neurotrauma. The Group was commissioned by the NIHR using UK aid from the UK Government (project 16/137/105). The views expressed in this article are those of the authors and are not necessarily those of the United Kingdom National Health Service, NIHR, or the United Kingdom Department of Health. F.N. is Founder and Chief Executive Officer of UpSurgeOn, viale Monza 347, 20126 Milan, Italy. R.M.G. serves as a National Institutes of Health Fogarty Global Health Fellow and Scholar. Research reported in this publication was supported by the 10.13039/100000061Fogarty International Center and 10.13039/100000025National Institute of Mental Health, of the 10.13039/100000002National Institutes of Health under Award Number D43 TW010543. The content is solely the responsibility of the authors and does not necessarily represent the official views of the National Institutes of Health.

## CRediT authorship contribution statement

**Sujit Gnanakumar:** Methodology, Investigation, Data curation, Writing - original draft. **Bilal Abou El Ela Bourquin:** Methodology, Investigation, Data curation, Writing - original draft. **Faith C. Robertson:** Methodology, Investigation, Data curation, Writing - original draft. **Davi J. Fontoura Solla:** Conceptualization, Data curation, Writing - review & editing. **Claire Karekezi:** Conceptualization, Writing - review & editing. **Kerry Vaughan:** Conceptualization, Writing - review & editing. **Roxanna M. Garcia:** Conceptualization, Writing - review & editing. **Fahd Derkaoui Hassani:** Conceptualization, Writing - review & editing. **Alexander Alamri:** Conceptualization, Writing - review & editing. **Julius Höhne:** Conceptualization, Writing - review & editing. **Nesrine Mentri:** Conceptualization, Writing - review & editing. **Martin Stienen:** Conceptualization, Writing - review & editing. **Tsegazeab Laeke:** Conceptualization, Writing - review & editing. **Luis Rafael Moscote-Salazar:** Conceptualization, Writing - review & editing. **Ahmed Nasser Al-Ahmari:** Conceptualization, Writing - review & editing. **Hosam Al-Jehani:** Conceptualization, Writing - review & editing. **Federico Nicolosi:** Conceptualization, Writing - review & editing. **Nicolás Samprón:** Conceptualization, Writing - review & editing. **P. David Adelson:** Conceptualization, Writing - review & editing. **Franco Servadei:** Conceptualization, Writing - review & editing. **Ignatius N. Esene:** Conceptualization, Data curation, Writing - review & editing. **Amro Al-Habib:** Conceptualization, Writing - review & editing. **Angelos G. Kolias:** Conceptualization, Methodology, Investigation, Data curation, Writing - review & editing.

## Supplementary data

Appendix 1 World Federation of Neurosurgical Young Neurosurgeons Survey.

Appendix 2 Frequency and Percentage of Survey Responses by Country.
