# Supplementary material for: The World Federation of Neurosurgical Societies Young Neurosurgeons Survey (Part I): Demographics, Resources, and Education
Source: World Neurosurg X. 2020 Oct;8:None. doi: 10.1016/j.wnsx.2020.100083 (PMC7573644; doi:10.1016/j.wnsx.2020.100083)
Supplement: Appendix 2 — Frequency and Percentage of Survey Responses by Country. [file mmc2.docx]

**Appendix 2. Frequency and Percentage of Survey Responses by Country**

| **Country** | **World Bank Category** | **Frequency** | **Percent** |
| --- | --- | --- | --- |
| Afghanistan | Low-income Economies | 2 | 0.2 |
| Albania | Upper-middle-income Economies | 1 | 0.1 |
| Algeria | Upper-middle-income Economies | 11 | 1.2 |
| Angola | Lower-middle-income Economies | 1 | 0.1 |
| Argentina | High-income Economies | 10 | 1 |
| Australia | High-income Economies | 2 | 0.2 |
| Austria | High-income Economies | 8 | 0.8 |
| Bahrain | High-income Economies | 1 | 0.1 |
| Bangladesh | Lower-middle-income Economies | 9 | 0.9 |
| Belarus | Upper-middle-income Economies | 2 | 0.2 |
| Belgium | High-income Economies | 8 | 0.8 |
| Bolivia | Lower-middle-income Economies | 4 | 0.4 |
| Brazil | Upper-middle-income Economies | 14 | 1.5 |
| Bulgaria | Upper-middle-income Economies | 5 | 0.5 |
| Cameroon | Lower-middle-income Economies | 1 | 0.1 |
| Canada | High-income Economies | 3 | 0.3 |
| Chad | Low-income Economies | 1 | 0.1 |
| Chile | High-income Economies | 3 | 0.3 |
| China | Upper-middle-income Economies | 3 | 0.3 |
| Colombia | Upper-middle-income Economies | 11 | 1.2 |
| Costa Rica | Upper-middle-income Economies | 1 | 0.1 |
| Côte d'Ivoire | Lower-middle-income Economies | 2 | 0.2 |
| Croatia | High-income Economies | 4 | 0.4 |
| Cuba | Upper-middle-income Economies | 4 | 0.4 |
| Czech Republic | High-income Economies | 2 | 0.2 |
| Denmark | High-income Economies | 4 | 0.4 |
| Ecuador | Upper-middle-income Economies | 4 | 0.4 |
| Egypt | Lower-middle-income Economies | 24 | 2.5 |
| El Salvador | Lower-middle-income Economies | 2 | 0.2 |
| Ethiopia | Low-income Economies | 12 | 1.3 |
| Finland | High-income Economies | 7 | 0.7 |
| France | High-income Economies | 9 | 0.9 |
| Georgia | Lower-middle-income Economies | 1 | 0.1 |
| Germany | High-income Economies | 75 | 7.9 |
| Greece | High-income Economies | 29 | 3 |
| Guatemala | Upper-middle-income Economies | 5 | 0.5 |
| Honduras | Lower-middle-income Economies | 2 | 0.2 |
| Hong Kong (S.A.R.) | High-income Economies | 1 | 0.1 |
| India | Lower-middle-income Economies | 73 | 7.7 |
| Indonesia | Lower-middle-income Economies | 23 | 2.4 |
| Iran, Islamic Republic of... | Upper-middle-income Economies | 2 | 0.2 |
| Iraq | Upper-middle-income Economies | 10 | 1 |
| Ireland | High-income Economies | 3 | 0.3 |
| Israel | High-income Economies | 4 | 0.4 |
| Italy | High-income Economies | 41 | 4.3 |
| Jordan | Upper-middle-income Economies | 4 | 0.4 |
| Kenya | Lower-middle-income Economies | 8 | 0.8 |
| Kuwait | High-income Economies | 1 | 0.1 |
| Kyrgyzstan | Lower-middle-income Economies | 1 | 0.1 |
| Latvia | High-income Economies | 5 | 0.5 |
| Lebanon | Upper-middle-income Economies | 1 | 0.1 |
| Libyan Arab Jamahiriya | Upper-middle-income Economies | 7 | 0.7 |
| Lithuania | High-income Economies | 2 | 0.2 |
| Luxembourg | High-income Economies | 1 | 0.1 |
| Malaysia | Upper-middle-income Economies | 5 | 0.5 |
| Mali | Low-income Economies | 2 | 0.2 |
| Mauritius | Upper-middle-income Economies | 1 | 0.1 |
| Mexico | Upper-middle-income Economies | 18 | 1.9 |
| Mongolia | Lower-middle-income Economies | 1 | 0.1 |
| Montenegro | Upper-middle-income Economies | 1 | 0.1 |
| Morocco | Lower-middle-income Economies | 4 | 0.4 |
| Mozambique | Low-income Economies | 1 | 0.1 |
| Myanmar | Lower-middle-income Economies | 4 | 0.4 |
| Nepal | Low-income Economies | 7 | 0.7 |
| Netherlands | High-income Economies | 20 | 2.1 |
| Nicaragua | Lower-middle-income Economies | 2 | 0.2 |
| Nigeria | Lower-middle-income Economies | 17 | 1.8 |
| Norway | High-income Economies | 1 | 0.1 |
| Pakistan | Lower-middle-income Economies | 26 | 2.7 |
| Panama | High-income Economies | 1 | 0.1 |
| Peru | Upper-middle-income Economies | 9 | 0.9 |
| Philippines | Lower-middle-income Economies | 12 | 1.3 |
| Poland | High-income Economies | 5 | 0.5 |
| Portugal | High-income Economies | 12 | 1.3 |
| Qatar | High-income Economies | 1 | 0.1 |
| Republic of Moldova | Lower-middle-income Economies | 5 | 0.5 |
| Romania | Upper-middle-income Economies | 8 | 0.8 |
| Russian Federation | Upper-middle-income Economies | 11 | 1.2 |
| Rwanda | Low-income Economies | 1 | 0.1 |
| Saudi Arabia | High-income Economies | 28 | 2.9 |
| Serbia | Upper-middle-income Economies | 11 | 1.2 |
| Slovakia | High-income Economies | 1 | 0.1 |
| South Africa | Upper-middle-income Economies | 7 | 0.7 |
| Spain | High-income Economies | 19 | 2 |
| Sudan | Lower-middle-income Economies | 6 | 0.6 |
| Sweden | High-income Economies | 7 | 0.7 |
| Switzerland | High-income Economies | 21 | 2.2 |
| Syrian Arab Republic | Low-income Economies | 6 | 0.6 |
| Tajikistan | Low-income Economies | 1 | 0.1 |
| Thailand | Upper-middle-income Economies | 3 | 0.3 |
| The former Yugoslav Republic of Macedonia | Upper-middle-income Economies | 1 | 0.1 |
| Tunisia | Lower-middle-income Economies | 3 | 0.3 |
| Turkey | Upper-middle-income Economies | 67 | 7 |
| Uganda | Low-income Economies | 1 | 0.1 |
| Ukraine | Lower-middle-income Economies | 14 | 1.5 |
| United Kingdom of Great Britain and Northern Ireland | High-income Economies | 47 | 4.9 |
| United Republic of Tanzania | Low-income Economies | 2 | 0.2 |
| United States of America | High-income Economies | 45 | 4.7 |
| Uzbekistan | Lower-middle-income Economies | 5 | 0.5 |
| Venezuela, Bolivarian Republic of... | Upper-middle-income Economies | 1 | 0.1 |
| Vietnam | Lower-middle-income Economies | 5 | 0.5 |
| Yemen | Low-income Economies | 3 | 0.3 |
| **Total** |  | **953** | **100** |
